# Dietary Fibre Intake in Australia. Paper II: Comparative Examination of Food Sources of Fibre among High and Low Fibre Consumers

**DOI:** 10.3390/nu10091223

**Published:** 2018-09-04

**Authors:** Flavia Fayet-Moore, Tim Cassettari, Kate Tuck, Andrew McConnell, Peter Petocz

**Affiliations:** 1 Nutrition Research Australia, Level 13 167 Macquarie Street, Sydney 2000, Australia; tim@nraus.com (T.C.); kate@nraus.com (K.T.); andrew@nraus.com (A.M.); 2 Department of Statistics, Macquarie University, Sydney 2109, Australia; peter.petocz@maquarie.edu.au

**Keywords:** dietary fibre, food sources, dietary intake, National Nutrition Survey, Australia

## Abstract

Intakes of dietary fibre in Australia are lower than recommended. An understanding of food choices associated with fibre intake can help to inform locally relevant dietary interventions that aim to increase its consumption. This study aimed to profile the relationship between dietary choices and fibre intake of Australians. Using Day 1 data from the 2011–2012 National Nutrition and Physical Activity Survey (*n* = 12,153, ≥2 years), dietary fibre intake was classified by quartiles for children (2–18 years) and adults (≥19 years). Intakes of the Australian Dietary Guidelines (ADG) food groups were calculated, as well as the major, sub-major, and minor food groups from the Australian Food Composition Database. Each of these food groups provide a progressively greater level of detail. Associations with ADG food groups and major food groups were determined, and the leading sub-major and minor food group sources of fibre for low (Quartile 1) and high (Quartile 4) fibre consumers were profiled. Energy-adjusted intakes of wholegrain and/or high fibre but not refined grain (cereal) foods, vegetables, and fruit were positively associated, and discretionary foods negatively associated, with quartile of fibre intake (*p* < 0.001). The top three sub-major food group sources of fibre were regular breads, cereal mixed dishes, and ready-to-eat breakfast cereals in high fibre consumers and regular breads, cereal mixed dishes, and potatoes in low fibre consumers. White breads was the leading minor food group contributor in low fibre consumers, and apples and lower sugar wheat based breakfast cereal were the leading fibre contributors in high fibre consumers in children and adults, respectively. Higher intakes of wholegrain, fruits, and vegetables, and a lower discretionary intake were associated with higher fibre intake. Encouraging these foods as part of any public health intervention is likely to be effective for increasing dietary fibre intakes.

## 1. Introduction

The benefits of dietary fibre are well established. Increased fibre consumption may support regular laxation [1] and reduce the risk of cardiovascular disease [2], type 2 diabetes [3] and all-cause mortality [4]. Dietary recommendations worldwide encourage consumption of fibre-rich foods [1], including the Australian Dietary Guidelines (ADG), which recommends varieties of the grain (cereal) foods which are wholegrain or wholemeal partly because they provide more dietary fibre [5].

A recent review of studies worldwide, which included the 2007 Australian National Children’s Nutrition and Physical Activity Survey, reported that fibre intakes was lower than recommended in all adult and most childhood populations [6]. In the most recent 2011–2012 National Nutrition and Physical Activity Survey (NNPAS) in Australia, more than 1 in 2 children and more than 7 in 10 adults had fibre intakes below the Adequate Intake (AI) (14–28 g for children, 25 g for adult females, and 30 g for adult males), and more than 4 in 5 adults did not meet the Suggested Dietary Target to reduce chronic disease risk (28 g for women and 38 g for men) [7].

The Australian Bureau of Statistics (ABS) published the per capita contribution of the major and sub-major food groups to daily fibre intake from the 2011–2012 NNPAS [8]. Foods and beverages were divided into 24 major food groups and 132 sub-major food groups. Cereals and cereal products, which contains basic cereal and grain foods such as bread, pasta, rice, and breakfast cereals, was the leading major food group contributor to dietary fibre for both adults and children [8], but the majority of grain intake in children and adults is refined and lower in fibre [9]. Few studies have explored how intakes of fibre sources differ between high and low fibre consumers, and there are no published data in Australia. Among adults, a greater intake of the grain (cereal) food group as stipulated in the ADG was associated with higher fibre intakes [10], yet 67–76% of adults and 61–74% of children did not meet the recommended serves of grain (cereal) foods [9]. Among children and adolescents, those who consumed breakfast cereal for breakfast had a higher fibre intake than those who had a non-cereal breakfast or who skipped breakfast altogether [11].

Intakes of the other food groups that are sources of dietary fibre, such as fruit, vegetables and legumes/beans, are also lower than recommended. In the 2011–2012 NNPAS, 71–77% of adults and 54% of children and adolescents did not meet the recommended daily serves for fruit (0.5–2 serves for children and two serves for adults), and 95–97% of adults and >99% of children and adolescents did not meet the recommended serves for vegetables and legumes/beans (2–5.5 for children and 5–6 for adults) [9]. The intake of discretionary foods and beverages has been inversely associated with fruit and vegetable intake in Australian adults [12]. There is a need to measure the contribution of different foods to dietary fibre intake, including the differences between high and lower fibre consumers, and investigate which ADG food groups are associated with fibre intake so that evidence-based and country-specific recommendations can be made. Further, there is a need to understand how the intakes of discretionary foods, and grain foods that are refined/low fibre, contribute to total fibre intakes, and how they compare with the contribution of whole/high fibre grain foods. Understanding the foods and food groups that are associated with higher fibre intakes from nationally representative data in Australia will help to drive recommendations for increasing fibre intakes.

The aim of this study was to profile the relationship between food choices and fibre intakes of Australian adults and children. Data from the 2011–2012 NNPAS were analysed to compare the leading sources of fibre between low and high fibre consumers and to determine associations between fibre intake and levels of consumption of the ADG Five Food Groups [5].

## 2. Materials and Methods

### 2.1. Survey Methodology

The 2011–2012 NNPAS is a nationally representative survey carried out by the Australian Bureau of Statistics (ABS) and forms part of the 2011–2013 Australian Health Survey. Detailed dietary information and physical activity data were collected for the NNPAS during face-to-face interviews by trained interviewers from the ABS. Data were collected from 12,153 participants aged 2 years and over, 7735 of whom provided dietary data for a second day of recall by telephone interview. Participants were categorized as children and adolescents (children) (2–18 years, *n* = 2812) or adults (≥19 years, *n* = 9341). An Automated Multiple-Pass Method, developed by the Agricultural Research Service of the United States Department of Agriculture [13], was used to capture all foods and beverages consumed by respondents within the 24 h prior to the interview day. To maximize the sample size, only the first day of dietary recall was used for all analyses. The data were weighted to represent the Australian population with weightings provided by the ABS and normalized separately for children and adults so that the sum of the weights was equal to the sample size. The interview components of the survey were conducted under the Census and Statistics Act 1905 and ethics approval was not necessary. Further survey details are available online under the Australian Health Survey: Users’ Guide, 2011–2013 [14].

### 2.2. Dietary Intake

Data were analysed using the Australian Food Composition Database (AUSNUT) developed by Food Standards Australia New Zealand [15]. The nutrient content of over 5000 foods and beverages in the AUSNUT were largely determined from analysed values, but also from other methods including other country’s food composition tables and food labels. Foods and beverages in the AUSNUT were classified into food groups called major, sub-major and minor, each providing a greater level of detail about the food or beverage consumed and a greater number of foods and beverages included in each group. There are 24 major food groups which specify the key ingredient, for example “cereals and cereal products”. At the sub-major food groups level, there are 132 groups, which specify characteristics such as meat species or plant types, for example “regular breads, and bread rolls (plain/unfilled/untopped varieties)”. At the lowest level of classification, there are 515 minor food groups, which provide detail including the level of saturated fat, sugars content or fortification status, for example “breads, and bread rolls, white, mandatorily fortified”.

The Australian Guide to Healthy Eating accompanies the ADG and encourages the consumption of the Five Food Groups [5]: grain (cereal) foods, mostly wholegrain and/or high cereal fibre varieties (grain (cereal) foods); vegetables and legumes/beans (vegetables); fruit; milk, yoghurt, cheese and/or their alternatives (mostly reduced fat) (dairy and alternatives); and lean meat and poultry, fish, eggs, tofu, nuts and seeds and legumes/beans (meats and alternatives). Grain (cereal) foods were further categorized as either wholegrain and/or higher fibre grain (cereal) (wholegrain), or refined and lower fibre grain (cereal) (refined grain). Wholegrain was coded by the ABS and defined as those products that retain all of the main biological components of the grain in the same relative proportions as the intact grain, irrespective of fibre content. Refined grain was defined by the ABS as core grain foods that were also not high in fibre. Major, sub-major, and minor food groups were composed of both wholegrain and refined grain foods or contained a mixture of both. For example, the major food group cereal and cereal products contains almost 500 foods, approximately half of which are wholegrain and half refined grain, whereas over 90% of the foods in the major food group cereal based products and dishes are refined grain. At the sub-major food group level, just over half the foods in regular breads were wholegrain, about 70% of ready-to-eat breakfast cereals were wholegrain, while almost all the foods in cereal mixed dishes contained refined grain and were not wholegrain. Discretionary food and beverage groups are defined as foods and drinks not necessary for a nutritious diet, and are high in saturated fat, added sugars, added salt, or alcohol and low in fibre [16].

### 2.3. Under-Reporting

Participants aged 10 years and over were classified as under-reporters or not under-reporters based on the Goldberg cut-off limit of 0.9 for the ratio of reported energy intake to predicted basal metabolic rate [17].

### 2.4. Statistical Analyses

The daily amount of each food and beverage consumed in the survey was measured in grams and kJ for each respondent. Total daily macro- and micronutrient intakes were calculated for each respondent, including total daily fibre intake, in grams per day. Children and adults were classified by quartile of their fibre intake, with Quartile 1 (Q1) defined as low fibre consumers and Quartile 4 (Q4) as high fibre consumers. Mean daily energy and fibre intakes were calculated for each quartile, as well as mean age and the proportion of the quartile that was female and that were under-reporters. General linear models using quartile of fibre intake as a factor and energy intake as a covariate were used to calculate the energy-adjusted marginal mean serves of each of the Five Food Groups and discretionary foods and beverages (1 serving = 600 kJ) by quartile of fibre intake. For the major food groups, the unadjusted mean fibre intake and the percent contribution of the food group to total daily fibre were calculated, as well as the total intake in grams of the food group. For both the sub-major and minor food groups, the proportion of people consuming the food group, the median daily intake in grams among consumers of the food group, and the percent contribution of the food group to total fibre intake were calculated. *p*-values for means by quartile of fibre intake were tested by ANOVA and post hoc comparisons between quartiles were tested using the Bonferroni Correction. *p*-values for the prevalence of females and under-reporters in each quartile were derived using Chi-square tests.

The statistical package IBM SPSS version 23.0 (IBM Corp., Armonk, NY, USA) was used for all analyses. Due to the large sample sizes, *p*-values < 0.001 were treated as significant.

## 3. Results

### 3.1. Profiles of High and Low Fibre Consumers

Among children, high fibre consumers were more likely to be male and older than low fibre consumers (Table 1). High fibre consumers had an energy intake that was nearly double that of low fibre consumers (10.6 ± 3.6 MJ vs. 5.7 ± 2.2 MJ) and a fibre intake more than three times higher than that of low fibre consumers (32.4 ± 8.1 g, vs. 9.6 ± 2.5 g). Fibre density increased with fibre intake, from 1.9 ± 0.7 g/MJ among low fibre consumers to 3.3 ± 1.0 g/MJ among high fibre consumers. Among children aged >10 years, almost half (47.8%) of low fibre consumers were under-reporters, compared to 2.9% of high fibre consumers.

Among adults, high fibre consumers were also more likely to be male than female, and there was no relationship with age (Table 1). High fibre consumers had a fibre intake almost four times higher than low fibre consumers (39.6 ± 10.8 g vs. 10.2 ± 3.0 g) and an energy intake that was almost double that of low fibre consumers (11.3 ± 3.8 MJ vs. 6.3 ± 2.7 MJ). Among high fibre consumers, fibre density was also double that of low fibre consumers (3.8 ± 1.4 g/MJ vs. 1.9 ± 1.3 g/MJ). Almost half (46.5%) of low fibre consumers were under-reporters, compared to 3.6% of high fibre consumers.

Among both children and adults, high fibre consumers had a lower daily intake of total and saturated fat, and added and free sugars, compared to low fibre consumers (Table S1). High fibre consumers had a higher daily intake of carbohydrates and all micronutrients analysed apart from sodium, niacin, and, among children, calcium.

### 3.2. Relationship of Fibre Intake with Intake of the Five Food Groups and Discretionary Foods and Beverages

**Figure 1:**
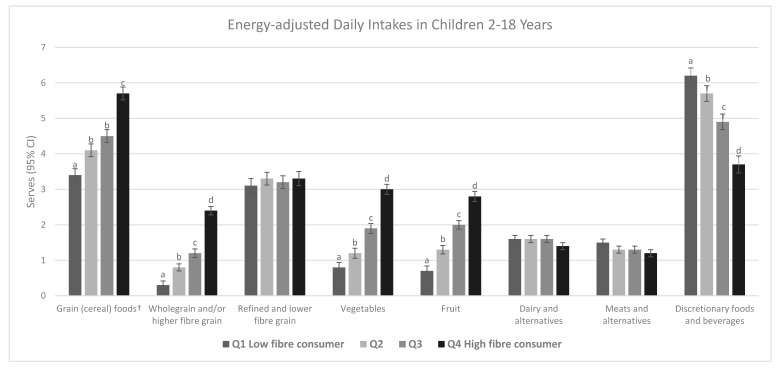
Energy-adjusted mean daily serves among children aged 2–18 years, from the Australian Dietary Guidelines five food groups and discretionary foods and beverages by quartile of fibre intake. a, b, c, d denote significant difference between quartiles (*p* < 0.001). Marginal mean serves from general linear models adjusted for energy intake. ^†^ Includes wholegrain and/or higher fibre grain, and refined and lower fibre grain.

**Figure 2:**
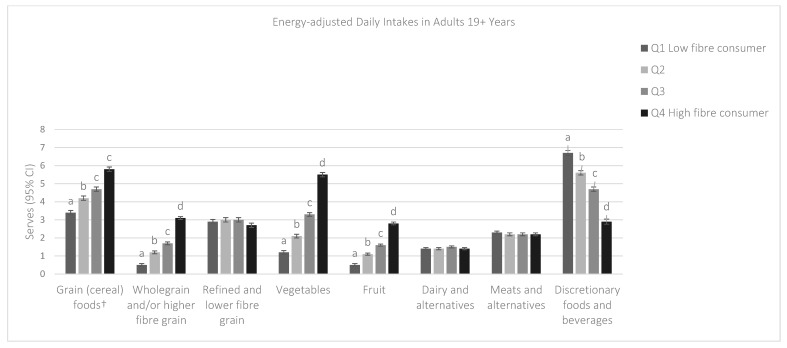
Energy-adjusted mean daily serves among adults 19 years and over from the Australian Dietary Guidelines five food groups and discretionary foods and beverages by quartile of fibre intake. a, b, c, d denote significant difference between quartiles (*p* < 0.001). Marginal mean serves from general linear models adjusted for energy intake. ^†^ Includes wholegrain and/or higher fibre grain, and refined and lower fibre grain.

The mean daily intakes of the Five Food Groups and discretionary foods and beverages adjusted for energy intake and categorized by quartile of fibre intake are shown in Figure 1 and Figure 2. Among both children and adults, as fibre intake increased, so did the energy-adjusted intake of the grain (cereal) food, fruit, and vegetable food groups, whilst discretionary intake decreased (*p* < 0.001). High fibre consumers had an additional two serves of grain (cereal) foods compared to low fibre consumers for both children (5.7 serves vs. 3.4 serves) and adults (5.8 serves, vs. 3.4 serves); an additional two serves of fruit; and four times the number of daily vegetables serves. Refined grain intake was similar across fibre quartiles for both adults and children, but high fibre consumers had eight times the number of daily serves of wholegrain compared to low fibre consumers in children, and more than six times as many serves in adults. Among both children and adults, discretionary intake was more than 40% lower among high fibre consumers compared to low fibre consumers. There were no differences in intakes of dairy and alternatives or meat and alternatives. 

### 3.3. Relationship of Fibre Intake with Food Choices: Major Food Groups

High fibre consumers had a greater mean fibre intake from all major food groups measured compared to low fibre consumers (Table 2). Among both children and adults, almost 30% of total daily fibre came from cereals and cereal products, which are largely comprised of core foods and wholegrains, and between 12% and 22% came from cereal based products and dishes, which are mostly discretionary or refined and lower fibre grains. Among adults, low fibre consumers had a higher proportion of total daily fibre from cereal based products and dishes compared to high fibre consumers (19.3% vs. 11.8%, respectively). For children, there was no significant difference in the contribution of cereals and cereal products or in the contribution of cereal based products and dishes to daily fibre across quartiles. High fibre consumers had double the intake of cereals and cereal products compared to low fibre consumers among children and adults, whereas for cereal based products and dishes there was no difference in intake between high and low fibre consumers. Among both adults and children, high fibre consumers had a greater contribution than low fibre consumers from non-grain fibre sources including fruit products and dishes, and legume and pulse products and dishes, and, among adults only, from nut and seed products. High fibre consumers had more than ten times the intake of fruit products and dishes compared to low fibre consumers. High fibre consumers also had more than double the intake of vegetable products and dishes among children and more than four times the intake among adults. 

### 3.4. Relationship of Fibre Intake with Food Choices: Sub-Major Food Groups

Among children, there were differences and similarities in the sub-major food groups that contributed the most fibre in low and high fibre consumers (Table 3). Both high and low consumers featured regular breads and cereal mixed dishes as the top two fibre contributors, with regular breads having the highest prevalence of consumers. The top three to five food groups were identical among high and low consumers, albeit in a different order, and included potato; ready-to-eat breakfast cereals; and pome fruit. Poultry mixed dishes and grain-based food groups including pastries and flours and other cereal grains were among the top sources of fibre in low but not high fibre consumers. In contrast, fruit and vegetable food groups including carrot and similar root vegetables; citrus fruit; and fruit and vegetable juices, and drinks were top fibre contributors in high but not in low fibre consumers. For all food groups that were in the top ten fibre contributors in both low and high fibre consumers, there was a higher prevalence of consumers among high fibre consumers. For certain food groups, there were large differences in the contribution to fibre intake between high and low fibre consumers despite similar total intakes of the food group. Regular breads contributed double the fibre among high compared to low fibre consumers, but the daily intake was similar (76.0 g compared to 64.0 g, respectively). Among consumers of ready-to-eat breakfast cereals, high consumers had double the daily intake of low fibre consumers, but the fibre contribution from the food group was four times higher.

Among adults, some food groups were similar and others different among the top fibre sources for both high and low fibre consumers. Both high and low consumers featured regular breads; cereal mixed dishes; ready-to-eat breakfast cereals; and potatoes in the top five fibre contributors. Regular breads was the top fibre contributor for low fibre consumers, and ready-to-eat breakfast cereals the top fibre contributor for high fibre consumers. Top ten fibre sources for low consumers included pastries, coffee and coffee substitutes, meat mixed dishes, and other vegetables and vegetable combinations, but these did not feature among high fibre consumers (Table 4). Fruit and vegetable food groups including soup, homemade; other fruiting vegetables; carrot and similar root vegetables; and tropical and subtropical fruit were among the top sources of fibre in high fibre consumers, but not low fibre consumers. High fibre consumers were more likely to consume these food groups than low fibre consumers and had higher daily intakes. Similar to children, regular breads had the highest prevalence of consumers among both low (55.0%) and high fibre consumers (70.3%). The daily intake of regular bread was 90 g among high fibre consumers compared to 66 g among low fibre consumers. Ready-to-eat breakfast cereals was consumed by 50.3% of high fibre consumers, with a daily intake of 70 g and 7.9 g of fibre, compared to 19.8% of low fibre consumers and a daily intake of 34 g and 1.9 g of fibre.

### 3.5. Relationship of Fibre Intake with Food Choices: Minor Food Groups

Minor food groups provide further information about the top food within the sub-major food groups. Among children, white breads, fortified and unknown fortification contributed the most to daily fibre intakes in low fibre consumers, and apples (part of the pome fruit sub-major food group) was the top fibre contributor among high fibre consumers (7.5%) followed by savory pasta and sauce dishes (Table 5). The main fibre contributors among low fibre consumers were white bread, potato and potato products, lower sugar wheat breakfast cereal, pasta, pastries and fruit such as apples and bananas. In high consumers, the main contributors were bread, including a mixed grain variety, fruit including apples and pears, potato products (not potatoes alone), carrots, pasta and instant noodles and lower sugar wheat breakfast cereal. The daily intakes of food groups in common were larger among high consumers compared to low consumers.

Among adults, white bread was the top fibre contributor in low fibre consumers (6.7%) but white bread was not in the top ten for high fibre consumers, who got more fibre from mixed grain bread (Table 6). Other food groups that were popular and in the top ten for low fibre consumers included potato products; salads, vegetable based; coffee, prepared with water; and savory pastry products, and none of these featured in the top ten for high fibre consumers. The top two sources of fibre for high consumers were both breakfast cereals (lower sugar wheat; and mixed grain with fruit and/or nut breakfast cereal), whereas breakfast cereals did not feature among the top fibre sources for low consumers. Vegetable soup, vegetables and sauce, and pears also contributed high amounts of fibre in high but not low fibre consumers. Savory pasta dishes were popular among both high and low fibre consumers and were in the top three fibre contributors.

## 4. Discussion

This study analysed food group intake of high and low fibre consumers and the association with total fibre intakes from nationally representative data in Australia. High fibre consumers tended to be male and had a greater energy intake than low fibre consumers. They also had greater intakes of three of the ADG Five Food Groups—grain (cereal) foods, fruits, and vegetables—and a lower intake of discretionary foods. The fibre intakes of low and high fibre consumers came from similar sources, in particular, breads, breakfast cereals, cereal dishes, pome fruit, and potatoes. Higher fibre consumers ate more of these foods and tended to make choices more aligned with Australian Dietary Guidelines, namely choosing higher fibre options such as mixed grain breads over white bread. 

High fibre consumers’ diets were characterized by a higher intake of grain (cereal) foods, fruits and vegetables. These food groups were the major sources of dietary fibre among both high and low fibre consumers, with cereal and cereal products contributing almost 30% of total daily fibre in all groups, and no other food groups contributing more than 20%. The importance of these food groups, particularly grain foods, in contributing to dietary fibre is in line with nutrition surveys globally. In a recent global review, grain products were the largest source of fibre in all countries, providing 32–49% of fibre in adults, followed by vegetables (12–21%), potatoes (6–19%) and fruit (8–23%). A higher energy-adjusted intake of these food groups has also been positively associated with fibre intake in USA children aged 2–5 years [18]. These findings are relevant given over 60% of adults have usual intakes of grain (cereal) foods less than recommended by the ADG [9]. Further, at least two in three Australians did not meet the recommended serves for fruits, and more than 95% consumed less than the recommended serves of vegetables [9]. Our findings highlight the importance of encouraging individuals to eat in accordance with national dietary guidelines to help reach adequate fibre intakes.

A previous analysis in Australian adults showed that a greater intake of the grain (cereal) food group was associated with higher fibre intakes [10], but the Australian Dietary Guidelines specify that cereal (grains) should be mostly wholegrain and/or high in fibre. We found that intake of whole and/or high fibre grains was strongly associated with greater daily fibre intake, where high fibre consumers had eight times as many wholegrain serves as low fibre consumers in children, and more than six times as many serves in adults. This is in line with previous findings from the UK, where children and adults who consumed more wholegrain had a higher total fibre intake [19]. In the USA, individuals in the highest tertile of wholegrain intake were 59–76 times more likely to fall in the highest fibre tertile, compared with those with no wholegrain intake [20]. The importance of wholegrain foods for increasing fibre intake is further supported by dietary intervention studies that showed an increase in wholegrain intake resulted in a subsequent increase in fibre intake [21,22]. This may be because wholegrains contain around 80% more fibre than refined grains [23], and can provide at least twice as much fibre than fruits and vegetables per 100 g [24].

In contrast, a higher intake of refined or lower fibre grain foods was not associated with a higher intake of fibre in this study. Our findings extend on previous research on the positive association of core grain foods and total fibre intake in Australian adults [10] to demonstrate that this association is specific to the whole and/or high fibre grain foods. In the Baltimore Longitudinal Study on Ageing, an open prospective cohort study, the intake of refined grains was inversely related to intake of whole grains and the percentage of energy from fibre [25]. The role of refined grains for increasing fibre may depend on their definition, whether intake is energy adjusted, and their associations with diet. Further, while refined grains were a major source of fibre, they may inadvertently contribute to the low fibre intakes by replacing potentially higher-fibre wholegrain foods.

High fibre consumers had lower intakes of discretionary energy and at the same time, higher intakes of three of the recommended Five Food Groups—grain (cereal) foods, vegetables, and fruit—which are all known sources of fibre. This inverse relationship with fibre and discretionary is expected, as discretionary foods and beverages are low in fibre by definition [16]. The other food groups; dairy and alternatives, and meats and alternatives, are also lower in fibre, but these food groups were not inversely associated with dietary fibre intake and rather had no association. This may be because these foods may be consumed with both high and low fibre foods. For example, milk is associated with breakfast cereal consumption [26] which can be high and low in fibre, and meat can accompany both starchy and non-starchy vegetables [27]. Further, we found that the proportion of fibre from the cereal based products and dishes food group, which provided almost 20% of total fibre intake in low fibre consumers, was inversely associated with fibre intake in adults. This food group is made up largely of discretionary foods, including sweet biscuits, pastries, cakes, and muffins; and refined core grain foods, such as savory biscuits (e.g., crackers) and cereal mixed dishes (e.g., sandwiches, pasta and rice dishes). While the vast majority of foods in the cereal based products and dishes food group are refined grain, and more than half are discretionary, only about half the foods in the cereal and cereal products group are refined grain, and almost none are discretionary. Our findings raise the possibility that whilst discretionary foods and beverages can provide some fibre, they may be a contributing factor to the low fibre intakes in Australia by displacing the intake of other fibre-rich foods and are a marker of poorer diet quality. A previous analysis of the 2011–2012 NNPAS in Australia found that discretionary foods and beverages were inversely associated with total fruit and vegetable consumption [12]. Similarly, in the USA, a high intake of energy-dense, nutrient-poor foods was associated with a low intake of core foods [28]. A modeling study in Australian adults found that removing discretionary foods from the diet would decrease total fibre intake, and that the substitution of discretionary with core foods would increase total fibre intake [29].

A greater intake of legume and pulse products and dishes, and nut and seed products and dishes was positively associated with higher fibre intakes, but their contribution to total fibre was low, even in high fibre consumers (<3%). This is not surprising given that consumption of these foods is generally low and infrequent. In the 2011–2012 NNPAS, seed and nut products and dishes were consumed by 17% of all Australians, and legume and pulse products and dishes were consumed by only 4.5% [8].

The sub-major food groups that contributed the most fibre were similar among high and low fibre consumers. These included breads, ready-to-eat breakfast cereals, cereal mixed dishes (e.g., sandwiches, pasta and rice dishes), pome fruit (e.g., apples and pears) and potatoes. This is in line with the diets of children and adults from the USA [30,31,32], where the top fibre sources include potatoes, bread, fruit and ready-to-eat breakfast cereals. These food groups may be relevant targets to increase fibre intake in Australians, with a focus on those that are non-discretionary and higher in fibre, alongside other sources of dietary fibre that are under-consumed, such as fruits, vegetables, and legumes.

This current analysis also provides insights into how the diet of high and low fibre consumers differs by comparing the leading minor food group sources of fibre, which have specific details about the types of foods consumed. Ready-to-eat breakfast cereals contributed the most fibre among high fibre consumers in adults. While high consumers had double the intake that low consumers had, they consumed four times as much fibre. This suggests that high fibre consumers were not only consuming more ready-to-eat breakfast cereals, but more importantly, that they were consuming cereals that were more fibre dense. A systematic review of studies in adults and children reported that breakfast cereal consumers have higher fibre intakes than those who do not consume breakfast cereals [26]. In Australia, breakfast cereals have been shown to be a leading source of total dietary fibre (9.1%) [8], cereal fibre (19–24.6%) [33] and wholegrain (~36%) intakes [34], and among Australian children, breakfast cereal consumers had a higher fibre intake [11,35] and were more likely to meet the fibre AI [35] compared to non-cereal consumers. Not all breakfast cereals are nutritionally comparable and breakfast cereals that are higher in fibre and lower in added sugars should be encouraged, to align with the ADG.

Regular breads were among the top two leading fibre contributors in high and low fibre consumers, and the leading fibre contributor among children for both high and low fibre consumers. The type of bread was also an important determinant of its contribution to fibre, with mixed grain bread a top contributor among high consumers, but not among low consumers. Similarly, national surveys in the USA [30], NZ [36], the UK [37] and Belgium [38] reported bread to be the largest source of fibre. In the National Diet and Nutrition Survey Rolling Programme in the UK, intake of all breads, and wholemeal bread, was positively associated with wholegrain intake, but intake of white bread was inversely associated with wholegrain intake [19]. This raises the possibility that a high intake of white bread, whilst being the leading fibre contributor in low fibre consumers, may also be a marker of a low intake of wholegrains, and of wholegrain bread specifically. The diets of high fibre consumers in our study were also characterized by a large difference in the median fibre obtained from regular breads and ready-to-eat breakfast cereals despite a proportionally smaller difference in the daily intake (grams) of the food group consumed compared to low fibre consumers. This may be because unlike fruits and vegetables, there is a large variation in the fibre content of common breads (~2.5–7 g/100 g) and particularly ready-to-eat breakfast cereals (~1–30 g/100 g) [39], with the capacity for these foods to be fibre-dense (grams of fibre/grams of food). Encouraging fibre-dense breads, including high fibre white bread, and higher fibre breakfast cereals may be useful to help increase fibre intakes.

A key finding of our study was that discretionary foods did not make a significant contribution to fibre intakes among high consumers. The leading fibre contributors consisted almost entirely of foods from the Five Food Groups for high fibre consumers, whereas among low consumers, potato products (e.g., fries, wedges, and hash browns) in adults and savory pastry products (e.g., dumplings, pies, sausage rolls, and spring rolls) in children, which are predominantly discretionary, were in the top ten fibre contributors among low fibre consumers, but not among the top ten for high fibre consumers. 

Strengths of our study include the use of a large nationally representative sample of children and adults, making our results generalizable to the Australian population. We included the median fibre and total daily intake for sub-major and minor food groups among consumers of these foods, to determine if the differences in fibre intake between high and low consumers were related to the quantities of food eaten or the differences in the fibre content of the foods consumed. Further, we adjusted for the differences in energy intake in our analysis of the associations with ADG food groups. Limitations should also be considered. We used one day of dietary recall to increase the sample size, which may not reflect usual intakes. As with other nutrition surveys, dietary fibre intake from the 2011–2012 NNPAS was affected by under-reporting [14], and we found a higher proportion of under-reporting and a lower energy intake among low fibre consumers compared to high fibre consumers. This may be partly due to some low dietary fibre consumers actively dieting and purposely reducing total energy intake, and not under-reporting as predicted by the EI: BMR ratio. The ABS coding of food groups is not designed with respect to fibre intake. For example, “breads, and bread rolls, white, mandatorily fortified” and “breads, and bread rolls, white, not stated as to fortification” are coded separately, despite no meaningful differences in fibre content. Comparison of the different food groups should be considered with the specific definitions in mind. Our analysis did not consider the type (e.g., soluble vs. insoluble) or source (e.g., cereal vs. fruit vs. vegetable) of fibre, although such analyses require assumptions due to the limitations in food composition databases. Further, there are currently no established intake guidelines that differentiate between fibre types or sources. Finally, data are cross-sectional, precluding causal relationships.

## 5. Conclusions

Increased intakes of grain (cereal) foods, specifically whole/high fibre, and fruits and vegetables, and a lower intake of discretionary foods and beverages, were associated with higher fibre intake, in line with the Australian Dietary Guidelines. Cereal (grain) foods were the top sources of fibre in the Australian diet, and high consumers had greater intakes of wholegrain and higher fibre varieties. Similar food groups contributed to fibre intakes among high and low consumers, but high consumers ate more of these foods and chose varieties that were more fibre dense. Results from this analysis can inform the development of food based dietary interventions to facilitate increased fibre intakes, which are currently inadequate.

## Figures and Tables

**Table 1:** Total fibre intake and demographic characteristics by quartiles of dietary fibre intake.

	Children (2–18 Years)					Adults (≥19 Years)				
	Low Fibre Consumer Q1	Q2	Q3	High Fibre Consumer Q4	*p* Value	Low Fibre Consumer Q1	Q2	Q3	High Fibre Consumer Q4	*p* Value
Range dietary fibre intake (g)	0–13.2	13.2–18.2	18.2–25.0	25.0–93.2		0–14.3	14.3–20.7	20.7–28.7	28.7–113.8	
Dietary fibre intake (g), median (IQR)	10.0 [7.8, 11.6]	15.5 [14.4, 16.8]	21.2 [19.6, 22.9]	29.7 [26.9, 35.4]		10.8 [8.4, 12.6]	17.5 [16.0, 19.0]	24.1 [22.2, 26.3]	36.4 [31.9, 44.0]	
Dietary fibre intake (g), mean (SD)	9.6 ^a^ (2.5)	15.6 ^b^ (1.4)	21.3 ^c^ (1.9)	32.4 ^d^ (8.1)	<0.001	10.2 ^a^ (3.0)	17.5 ^b^ (1.8)	24.3 ^c^ (2.3)	39.6 ^d^ (10.8)	<0.001
Dietary fibre density (g/MJ), mean (SD)	1.9 ^a^ (0.7)	2.4 ^b^ (0.8)	2.7 ^c^ (0.8)	3.3 ^d^ (1.0)	<0.001	1.9 ^a^ (1.3)	2.5 ^b^ (0.9)	3.0 ^c^ (1.0)	3.8 ^d^ (1.4)	<0.001
Energy intake (MJ), mean (SD)	5.7 ^a^ (2.2)	7.2 ^b^ (2.3)	8.5 ^c^ (2.5) ^c^	10.6 ^d^ (3.6)	<0.001	6.3 ^a^ (2.7)	7.8 ^b^ (2.8)	9.3 ^c^ (3.2)	11.3 ^d^ (3.8)	<0.001
Energy intake (MJ), median [IQR]	5.4 [4.0, 6.9]	7.0 [5.6, 8.7]	8.4 [6.6, 9.8]	10.1 [8.2, 12.1]		5.9 [4.3, 7.8]	7.4 [5.8, 9.4]	8.8 [6.9, 11.0]	10.6 [8.7, 13.4]	
Age (years), mean (SD)	9.3 ^a^ (5.3)	9.5 ^a^ (4.8)	10.0 ^a,b^ (4.7)	10.9 ^b^ (4.4)	<0.001	45.5 (17.8)	46.4 (17.3)	46.4 (17.4)	46.7 (17.4)	0.112
Female (within fibre quartile), %	57.0	53.3	48.2	37.5	<0.001	59.8	54.5	50.9	37.2	<0.001
Under-reported * (within fibre quartile), %	47.8	17.8	7.1	2.9	<0.001	46.5	24.6	12.7	3.6	<0.001

Abbreviations: Q, quartile; IQR, inter-quartile range; SD, standard deviation; MJ, Megajoules. Different superscripts a, b, c, d denote significant difference between groups (*p* < 0.001). * Participants aged 10 years and older were classified as under-reporters based on the Goldberg cut-off limit of an energy intake to basal metabolic rate ratio of 0.9 [17].

**Table 2:** Fibre contribution from major food groups by quartile of fibre intake.

Major Food Group	Children (2–18 Years)						Adults (≥19 Years)				
	Low Fibre Consumer Q1	Q2	Q3	High Fibre Consumer Q4	*p* Value	Low Fibre Consumer Q1		Q2	Q3	High Fibre Consumer Q4	*p* Value
Cereals and cereal products *											
Fibre intake (g), mean (SD)	2.9 ^a^ (2.3)	4.7 ^b^ (3.1)	5.8 ^c^ (3.7)	9.7 ^d^ (6.8)	<0.001	3.0 ^a^ (2.6)		5.0 ^b^ (3.6)	6.8 ^c^ (4.6)	11.8 ^d^ (8.4)	<0.001
Proportion of total daily fibre (%), mean (SD)	30.3 (23.2)	30.0 (19.7)	2 7.4 (17.5)	30.0 (18.9)	0.019	28.8 (24.3)		28.6 (20.0)	27.8 (18.6)	29.8 (19.1)	0.011
Energy-adjusted intake (g), mean (95% CI)	109.8 ^a^ (98.1–121.5)	144.9 ^b^ (131.1–155.6)	158.3 ^b^ (147.5–169.1)	215.6 ^c^ (203.8–227.4)	<0.001	114.8 ^a^ (107.4–122.3)		141.9 ^b^ (134.9–148.9)	164.5 ^c^ (157.5–171.5)	234.0 ^d^ (226.5–241.5)	<0.001
**Cereal based products and dishes ^†^**											
Fibre intake (g), mean (SD)	1.9 ^a^ (2.1)	3.4 ^b^ (3.3)	4.1 ^b^ (3.9)	5.7 ^c^ (5.9)	<0.001	2.0 ^a^ (2.7)		3.1 ^b^ (3.6)	3.9 ^c^ (4.6)	4.5 ^d^ (6.4)	<0.001
Proportion of total daily fibre (%), mean (SD)	19.3 (21.7)	22.0 (21.7)	19.3 (18.5)	17.6 (18.4)	0.001	19.3 ^a^ (25.4)		17.5 ^a,b^ (20.7)	15.8 ^b^ (18.9)	11.8 ^c^ (16.0)	<0.001
Energy-adjusted intake (g), mean (95% CI)	167.6 (152.5–182.7)	191.5 (177.5–205.4)	194.5 (180.5–208.5)	191.9 (176.6–207.2)	0.048	159.8 ^a,b^ (150.7–168.9)		174.7 ^a^ (166.1–183.3)	181.0 ^a^ (172.5–189.6)	148.2 ^b^ (139.0–157.3)	<0.001
**Fruit products and dishes**											
Fibre intake (g), mean (SD)	1.3 ^a^ (1.7)	2.6 ^b^ (2.7)	4.1 ^c^ (3.3)	6.1 ^d^ (5.4)	<0.001	1.0 ^a^ (1.7)		2.4 ^b^ (2.9)	3.5 ^c^ (3.7)	6.3 ^d^ (6.2)	<0.001
Proportion of total daily fibre (%), mean (SD)	12.8 ^a^ (17.0)	16.2 ^b^ (17.1)	19.0 ^b^ (15.2)	19.1 ^b^ (15.6)	<0.001	8.8 ^a^ (15.8)		13.4 ^b^ (16.5)	14.4 ^b,c^ (15.0)	15.9 ^c^ (14.6)	<0.001
Energy-adjusted intake (g), mean (95% CI)	30.0 ^a^ (17.4–42.6)	107.9 ^b^ (96.3–119.5)	190.3 ^c^ (178.6–202.0)	302.8 ^d^ (290.0–315.5)	<0.001		25.0 ^a^ (17.5–32.6)	101.5 ^b^ (94.4–108.6)	158.8 ^c^ (151.7–165.8)	285.2 ^d^ (277.6–292.8)	<0.001
**Vegetable products and dishes**											
Fibre intake (g), mean (SD)	1.3 ^a^ (1.8)	2.1 ^a^ (2.4)	3.1 ^b^ (3.6)	4.8 ^c^ (5.6)	<0.001		1.9 ^a^ (2.3)	3.2 ^b^ (3.4)	5.2 ^c^ (4.9)	8.0 ^d^ (8.3)	<0.001
Proportion of total daily fibre (%), mean (SD)	14.4 (21.7)	13.1 (21.7)	14.7 (18.5)	14.6 (18.4)	0.3		18.5^a^ (21.4)	18.1 ^a^ (19.2)	21.4 ^b^ (20.3)	20.2 ^a,b^ (19.5)	<0.001
Energy-adjusted intake (g), mean (95% CI)	65.4 ^a^ (54.9–75.9)	81.2 ^a^ (71.5–90.0)	110.9 ^b^ (101.2–120.6)	148.9 ^c^ (138.2–159.5)	<0.001		78.7 ^a^ (70.6–86.8)	125.2 ^b^ (117.6–132.9)	191.9 ^c^ (184.3–199.6)	289.9 ^d^ (281.7–298.0)	<0.001
**Legume and pulse products and dishes**											
Fibre intake (g), mean (SD)	0.0 ^a^ (0.3)	0.1 ^a^ (0.8)	0.2 ^a^ (1.0)	0.9 ^b^ (4.1)	<0.001		0.0 ^a^ (0.4)	0.2 ^a^ (1.2)	0.2 ^a^ (1.4)	1.2 ^b^ (4.7)	<0.001
Proportion of total daily fibre (%), mean (SD)	0.4 ^a^ (3.7)	0.6 ^a^ (5.0)	0.8 ^a^ (5.1)	2.4 ^b^ (9.9)	<0.001		0.4 ^a^ (4.0)	1.0 ^a^ (6.3)	0.9 ^a^ (5.8)	2.8 ^b^ (10.2)	<0.001
Energy-adjusted intake (g), mean (95% CI)	1.5 ^a^ (0.0–6.1)	2.4 ^a^ (0.0–6.6)	3.3 ^a^ (0.0–7.6)	19.3 ^b^ (14.6–23.9)	<0.001		0.0 ^a^ (0.0–1.6)	2.9 ^a^ (0.8–5.1)	5.0 ^a^ (2.8–7.2)	27.7 ^b^ (25.4–30.0)	<0.001
**Seed and nut products and dishes**											
Fibre intake (g), mean (SD)	0.1 ^a^ (0.3)	0.1 ^a^ (0.4)	0.2 ^a,b^ (0.6)	0.3 ^b^ (1.2)	<0.001		0.1 ^a^ (0.6)	0.3 ^a,b^ (1.1)	0.5 (1.3)	1.2 ^c^ (3.3)	<0.001
Proportion of total daily fibre (%), mean (SD)	0.5 (2.7)	0.5 (2.4)	0.8 (2.9)	0.7 (3.2)	0.062		1.1^a^ (5.2)	1.9 ^b^ (6.0)	1.9 (5.3)	2.8 ^c^ (7.2)	<0.001
Energy-adjusted intake (g) mean (95% CI)	2.6 (1.4–3.7)	1.4 (0.3–2.4)	2.5 (1.4–3.6)	2.8 (1.6–3.9)	0.262		3.8 ^a^ (2.9–4.8)	6.0 ^a^ (5.0–6.9)	6.2 ^a^ (5.3–7.1)	9.9 ^b^ (8.9–10.9)	<0.001

Abbreviations: Q, quartile; SD, standard deviation; CI, confidence interval. Different superscripts a, b, c, d denote significant difference between groups (*p* < 0.001). * Includes flours and other grains, breads, pasta and pasta products (without sauce), and breakfast cereals. ^†^ Includes sweet and savory biscuits, cakes, muffins, and desserts, pastries, cereal mixed dishes, and batter-based products.

**Table 3:** Leading sub-major food groups by contribution to dietary fibre intake in children aged 2–18 years.

	Low Fibre Consumer Q1					High Fibre Consumer Q4				
				Intake among Consumers of the Food Group					Intake among Consumers of the Food Group	
Rank	Sub-Major Food Group	Proportion of Total Daily Fibre, % *	Consumers, %	Daily Intake (g), Median [IQR]	Fibre (g), Median [IQR]	Sub-Major Food Group	Proportion of Total Daily Fibre, % *	Consumers,%	Daily Intake (g), Median [IQR]	Fibre (g), Median [IQR]
1	Regular breads ^1^	16.7%	60.3%	64.0 [40.0–91.0]	2.1 [1.8–3.9]	Regular breads ^1^	11.2%	76.0%	76.0 [64.0–130.0]	4.1 [2.1–6.2]
2	Cereal mixed dishes ^2^	11.1%	34.8%	163.7 [110.0–209.2]	2.7 [1.9–4.0]	Cereal mixed dishes ^2^	11.0%	46.2%	338.0 [196.0–468.0]	6.9 [3.5–10.3]
3	Potatoes	7.2%	33.7%	65.0 [28.5–122.5]	1.7 [0.9–2.9]	Ready-to-eat breakfast cereals ^3^	10.6%	54.9%	51.0 [34.0–81.0]	4.4 [2.0–8.6]
4	Ready-to-eat breakfast cereals ^3^	6.1%	35.9%	26.0 [17.0–34.0]	1.1 [0.6–2.3]	Pome fruit	9.8%	53.6%	164.0 [153.0–326.4]	3.8 [3.7–7.5]
5	Pome fruit	5.6%	18.1%	143.0 [82.0–164.0]	3.3 [1.9–3.8]	Potatoes	4.4%	34.5%	122.5 [68.7–203.0]	2.9 [1.8–4.7]
6	Poultry mixed dishes ^4^	3.3%	19.4%	114.0 [66.2–176.4]	1.1 [0.7–2.2]	Pasta and pasta products ^5^	3.4%	13.7%	250.0 [155.0–385.0]	5.1 [4.5–9.9]
7	Tropical and subtropical fruit	3.1%	13.0%	98.0 [74.0–111.0]	2.4 [1.8–2.5]	Carrot and similar root vegetables	2.7%	24.9%	61.0 [32.0–129.0]	2.2 [1.2–5.2]
8	Pasta and pasta products ^5^	2.6%	7.2%	145.0 [93.0–267.1]	3.7 [1.8–4.9]	Citrus fruit	2.4%	24.8%	131.0 [75.0–192.2]	1.8 [0.9–3.4]
9	Pastries	2.5%	9.8%	94.8 [51.5–175.0]	2.0 [0.9–3.6]	Tropical and subtropical fruit	2.3%	26.1%	98.0 [98.0–155.8]	2.4 [2.4–3.3]
10	Flours and other cereal grains ^6^	2.2%	13.9%	135.2 [45.6–219.1]	1.4 [0.5–2.3]	Fruit and vegetable juices, and drinks	2.3%	38.6%	313.5 [210.0–520.0]	0.6 [0.2–1.5]

Abbreviations: Q, quartile; IQR, inter-quartile range. * Calculated at overall level (fibre intake from the food group among all respondents/total daily fibre intake among all respondents). ^1^ Regular breads, and bread rolls (plain/unfilled/untopped varieties). ^2^ Mixed dishes where cereal is the major ingredient and includes foods such as sandwiches, burgers, pizzas, tacos, and pasta and rice dishes. ^3^ Breakfast cereals, ready to eat. ^4^ Mixed dishes where poultry or feathered game is the major component. ^5^ Pasta and pasta products (without sauce). ^6^ Flours and other cereal grains and starches.

**Table 4:** Leading sub-major food groups by contribution to dietary fibre intake in adults aged 19 years and older.

	Low Fibre Consumer Q1					High Fibre Consumer Q4				
				Intake among Consumers of the Food Group					Intake among Consumers of the Food Group	
Rank	Sub-Major Food Group	Proportion of Total Daily Fibre, % *	Consumers, %	Daily Intake (g), Median [IQR]	Fibre (g), Median [IQR]	Sub-Major Food Group	Proportion of Total Daily Fibre, % *	Consumers, %	Daily Intake (g), Median [IQR]	Fibre (g), Median [IQR]
1	Regular breads ^1^	17.2%	55.0%	66.0 [54.0–99.0]	2.8 [1.3–4.1]	Ready-to-eat breakfast cereals ^2^	12.3%	50.3%	70.5 [40.0–108.0]	7.9 [3.8–13.3]
2	Cereal mixed dishes ^3^	11.6%	29.7%	196.7 [129.8–312.0]	3.4 [2.1–5.6]	Regular breads ^1^	10.2%	70.3%	90.0 [64.0–138.0]	4.5 [2.9–7.6]
3	Potatoes	5.5%	23.9%	82.5 [39.6–132.1]	2.1 [1.1–3.3]	Cereal mixed dishes ^3^	7.2%	31.7%	350.0 [218.1–532.5]	7.5 [3.9–11.5]
4	Ready-to-eat breakfast cereals ^2^	4.5%	19.8%	34.0 [21.9–42.5]	1.9 [1.0–3.7]	Pome fruit	5.9%	40.8%	171.0 [164.0–263.0]	4.1 [3.8–7.3]
5	Vegetable dishes ^4^	3.9%	24.1%	65.0 [46.5–127.0]	1.3 [0.8–2.1]	Potatoes	4.0%	33.1%	172.0 [95.0–260.0]	3.6 [2.2–6.5]
6	Pastries	3.5%	11.7%	140.0 [78.6–175.0]	3.3 [1.8–4.2]	Vegetable dishes ^4^	3.8%	25.4%	155.0 [66.1–310.0]	2.9 [1.3–7.0]
7	Coffee and coffee substitutes	3.0%	53.6%	281.0 [200.0–500.0]	0.4 [0.1–0.8]	Soup, homemade ^5^	3.0%	10.4%	515.0 [319.2–909.0]	9.1 [5.4–14.9]
8	Meat mixed dishes ^6^	2.8%	11.5%	184.0 [79.9–282.5]	1.9 [0.9–3.7]	Other fruiting vegetables	2.9%	34.0%	79.5 [27.2–156.0]	1.9 [0.7–4.3]
9	Pome fruit	2.7%	7.6%	160.0 [139.0–173.0]	3.8 [2.9–4.2]	Carrot and similar root vegetables	2.8%	25.1%	78.0 [30.0–162.1]	2.9 [1.2–5.9]
10	Other vegetables and vegetable combinations	2.7%	14.4%	20.5 [8.8–71.3]	1.0 [ 0.3–2.9]	Tropical and subtropical fruit	2.8%	30.5%	111.0 [98.0–183.5]	2.7 [2.4–4.3]

Abbreviations: Q, quartile; IQR, inter-quartile range. * Calculated at overall level (fibre intake from the food group among all respondents/total daily fibre intake among all respondents). ^1^ Regular breads, and bread rolls (plain/unfilled/untopped varieties). ^2^ Breakfast cereals, ready to eat. ^3^ Mixed dishes where cereal is the major ingredient and includes foods such as sandwiches, burgers, pizzas, tacos, and pasta and rice dishes. ^4^ Dishes where vegetable is the major component and includes foods like vegetable curries and stir-fry, stuffed vegetables, and salads. ^5^ Soup, homemade from basic ingredients. ^6^ Mixed dishes where beef, sheep, pork or mammalian game is the major component.

**Table 5:** Leading minor food groups by contribution to dietary fibre intake in children aged 2–18 years.

	Low Fibre Consumer Q1					High Fibre Consumer Q4				
				Intake among Consumers of the Food Group					Intake among Consumers of the Food Group	
Rank	Minor Food Group	Proportion of Total Daily Fibre, %	Consumers, %	Daily Intake (g), Median [IQR]	Fibre (g), Median [IQR]	Minor Food Group	Proportion of Total Daily Fibre, % *	Consumers, %	Daily Intake (g), Median [IQR]	Fibre (g), Median [IQR]
1	White breads, fortified ^1^	6.5%	32.0%	54.0 [33.0–70.0]	1.8 [1.0–2.3]	Apples	7.5%	49.6%	164.0 [153.0–286.0]	3.8 [3.6–6.6]
2	White breads, unknown fortification ^2^	6.4%	26.3%	64.0 [64.0–64.0]	2.1 [2.1–2.1]	Savory pasta and sauce dishes ^3^	5.5%	19.8%	419.5 [312.0–598.0]	8.1 [5.9–11.9]
3	Apples	5.0%	17.1%	143.0 [82.0–164.0]	3.3 [1.9–3.8]	Breakfast cereal, wheat based, fortified, sugars ≤20 g/100 g	4.9%	21.7%	51.0 [34.0–68.0]	5.6 [3.7–9.3]
4	Potato products	4.1%	19.5%	57.0 [28.5–84.2]	1.7 [0.9–2.6]	Potato products	2.6%	16.3%	105.0 [68.0–195.5]	3.3 [2.1–5.9]
5	Savory pasta and sauce dishes ^3^	3.5%	12.3%	156.0 [98.0–208.0]	2.1 [1.4–4.0]	White breads, fortified ^1^	2.5%	29.6%	69.0 [54.0–113.3]	2.1 [1.8–3.8]
6	Breakfast cereal, wheat based, fortified, sugars ≤20 g/100 g	3.0%	9.9%	25.5 [17.0–34.0]	2.8 [1.9–3.7]	Instant noodles and noodle products, wheat based	2.4%	6.9%	385.0 [145.0–385.0]	9.9 [5.0–13.1]
7	Bananas	2.6%	11.4%	98.0 [74.0–98.0]	2.4 [1.8–2.4]	Pears	2.2%	10.0%	181.0 [168.5–218.0]	6.5 [5.5–7.9]
8	Savory pastry products ^4^	2.1%	6.9%	130.0 [78.0–175.0]	2.3 [1.8–3.9]	Carrots	2.2%	22.6%	60.0 [30.0–129.0]	2.0 [1.1–5.2]
9	Potatoes	2.1%	11.5%	70.0 [39.1–110.6]	1.7 [0.8–2.7]	Mixed grain bread, unknown fortification ^5^	1.8%	9.3%	72.0 [72.0–123.7]	4.5 [4.5–7.8]
10	Rice and rice grain fractions	2.0%	13.7%	134.0 [45.6–201.0]	1.4 [0.5–2.1]	White breads, unknown fortification ^2^	1.8%	21.1%	64.0 [64.0–82.0]	2.1 [2.1–2.6]

Abbreviations: Q, quartile; IQR, inter-quartile range. * Calculated at overall level (fibre intake from the food group among all respondents/total daily fibre intake among all respondents). ^1^ Breads, and breads rolls, white, mandatorily fortified. ^2^ Breads, and breads rolls, white, not stated as to fortification. ^3^ Savory pasta/noodles and sauce dishes, saturated fat ≤5 g/100 g. ^4^ Savory pastry products, pies, rolls and envelopes. ^5^ Breads, and bread rolls, mixed grain, not stated as to fortification.

**Table 6:** Leading minor food groups by contribution to dietary fibre intake in adults aged 19 years and over.

	Low Fibre Consumer Q1					High Fibre Consumer Q4				
				Intake among Consumers of the Food Group					Intake among Consumers of the Food Group	
Rank	Minor Food Group	Proportion of Total Daily Fibre, % *	Consumers, %	Daily Intake (g), median [IQR]	Fibre (g), Median [IQR]	Minor Food Group	Proportion of Total Daily Fibre, % *	Consumers, %	Daily Intake (g), Median [IQR]	Fibre (g), Median [IQR]
1	White breads, fortified ^1^	6.7%	30.6%	66.0 [49.1–81.0]	2.0 [1.7–2.8]	Breakfast cereal, wheat based, fortified, sugars ≤20 g/100 g	4.4%	19.0%	51.0 [34.0–84.0]	7.5 [3.7–11.2]
2	Savory pasta and sauce dishes	3.6%	8.4%	208.0 [130.0–338.0]	3.6 [2.1–6.2]	Breakfast cereal, mixed grain, with fruit and/or nuts	4.3%	15.1%	91.9 [60.0–141.0]	10.2 [6.6–15.2]
3	Potato products	2.9%	13.2%	57.0 [28.5–104.0]	1.8 [1.0–3.2]	Savory pasta and sauce dishes ^2^	4.0%	15.1%	468.0 [260.0–676.0]	8.8 [4.8–12.6]
4	White breads, unknown fortification ^3^	2.9%	12.1%	64.0 [64.0–70.0]	2.1 [2.1–2.6]	Apples	3.9%	34.1%	164.0 [159.8–188.0]	3.8 [3.7–4.3]
5	Salads, vegetable based	2.8%	18.2%	60.0 [40.0–100.8]	1.1 [0.8–2.0]	Potatoes	2.5%	21.8%	172.0 [104.0–250.0]	3.8 [2.3–5.8]
6	Coffee, prepared with water ^4^	2.6%	38.9%	220.0 [200.0–450.0]	0.4 [0.4–0.9]	Soup, vegetable only	2.1%	6.9%	252.5 [252.5–538.6]	3.4 [0.6–5.1]
7	Savory pastry products ^5^	2.4%	7.2%	175.0 [114.0–175.0]	3.6 [2.0–4.2]	Mixtures of two or more vegetables	2.1%	11.3%	171.0 [114.0–256.5]	6.6 [4.2–9.6]
8	Apples	2.2%	6.5%	160.0 [139.0–164.0]	3.7 [2.9–3.8]	Mixed grain breads, fortified ^6^	2.1%	14.8%	62.0 [56.0–90.0]	4.4 [3.9–6.7]
9	Mixtures of two or more vegetables	2.2%	6.7%	71.3 [34.2–142.5]	2.9 [1.4–5.0]	Vegetables and sauce	2.0%	5.5%	386.8 [278.3–580.5]	12.5 [8.6–20.1]
10	Potatoes	2.0%	8.6%	101.3 [52.8–152.2]	2.2 [1.3–3.6]	Pears	1.9%	10.9%	181.0 [171.0–218.0]	6.5 [5.5–7.9]

Abbreviations: Q, quartile; IQR, inter-quartile range. * Calculated at overall level (Fibre intake from the food group among all respondents/total daily fibre intake among all respondents). ^1^ Breads, and breads rolls, white, mandatorily fortified. ^2^ Savory pasta/noodles and sauce dishes, saturated fat ≤5 g/100 g. ^3^ Breads, and breads rolls, white, not stated as to fortification. ^4^ Coffee beverage, prepared with water. ^5^ Savory pastry products, pies, rolls and envelopes. ^6^ Breads, and bread rolls, mixed grain, mandatorily fortified.

## Supplementary Materials

The following are available online at http://www.mdpi.com/2072-6643/10/9/1223/s1, Table S1: Daily nutrient intakes by quartile of dietary fibre intake.
