# Supplementary material for: Dietary Fibre Intake in Australia. Paper II: Comparative Examination of Food Sources of Fibre among High and Low Fibre Consumers
Source: Nutrients. 2018 Sep 4;10(9):1223. doi: 10.3390/nu10091223 (PMC6163727; doi:10.3390/nu10091223)
Supplement: Supplementary file 1 [file nutrients-10-01223-s001.pdf]

Supplementary Table 1. Daily nutrient intakes by quartile of dietary fiber intake.

Nutrient	**Children. (2–18 years)**					**Adults. (19+ years)**				
	Low Fiber Consumer. Q1	Q2	Q3	High Fiber Consumer. Q4	*p* value	Low Fiber Consumer. Q1	Q2	Q3	High Fiber Consumer. Q4	*p* value
	Energy-adjusted nutrient intake, marginal mean (SE)					Energy-adjusted nutrient intake, marginal mean (SE)				
Protein (g)	76.3 (0.9)	74.6 (0.9)	74.8 (0.9)	74.1 (0.9)	0.439	90.1 (0.7)	91.1 (0.6)	91.9 (0.6)	90.6 (0.7)	0.243
Total fat (g)	76.4 ^{a} (0.6)	73.0 ^{b} (0.6)	66.5 ^{c} (0.6)	60.1 ^{d} (0.7)	<0.001	77.8 ^{a} (0.5)	75.3 ^{a,b} (0.5)	73.5 ^{b} (0.5)	68 ^{c} (0.5)	<0.001
Saturated Fat (g)	33.0 ^{a} (0.4)	31.5 ^{a} (0.3)	27.7 ^{b} (0.3)	23.8 ^{c} (0.4)	<0.001	31.0 ^{a} (0.2)	29.1 ^{b} (0.2)	27.6 ^{c} (0.2)	22.7 ^{d} (0.2)	<0.001
Carbohydrates (g)	226 ^{a} (2)	232 ^{a} (2)	243 ^{b} (2)	253 ^{c} (2)	<0.001	206 ^{a} (1)	219 ^{b} (1)	227 ^{c} (1)	250 ^{d} (1)	<0.001
Total sugars (g)	116 (1.7)	109 (1.6)	113 (1.6)	110 (1.8)	0.009	101 (1)	101 (1)	101 (1)	107 (1)	0.001
Added sugars (g)	77.3 ^{a} (1.5)	62.8 ^{b} (1.4)	53.6 ^{c} (1.4)	37.4 ^{d} (1.5)	<0.001	68.2 ^{a} (1.0)	57.0 ^{b} (0.9)	45.8 ^{c} (0.9)	30.3 ^{d} (1.0)	<0.001
Free sugars (g)	84.7 ^{a} (1.6)	69.9 ^{b} (1.4)	62.6 ^{b} (1.5)	46.9 ^{c} (1.6)	<0.001	74.2 ^{a} (1.0)	63.6 ^{b} (1.0)	53.3 ^{c} (1.0)	39.2 ^{d} (1.0)	<0.001
Sodium (mg)	2363 (36)	2369 (33)	2222 (34)	2312 (37)	0.009	2397 (25)	2465 (24)	2479 (24)	2359 (25)	0.001
Calcium (mg)	782 (16)	797 (15)	804 (15)	828 (16)	0.305	726 ^{a} (9)	781 ^{b} (8)	819 ^{b} (8)	879 ^{c} (9)	<0.001
Iron (mg)	8.0 ^{a} (0.2)	8.9 ^{b} (0.1)	9.8 ^{c} (0.1)	12.1 ^{d} (0.2)	<0.001	8.6 ^{a} (0.1)	9.8 ^{b} (0.1)	11.5 ^{c} (0.1)	14.6 ^{d} (0.1)	<0.001
Thiamin (mg)	1.2 ^{a} (0.0)	1.6 ^{b} (0.0)	1.6 ^{b} (0.0)	2.1 ^{c} (0.0)	<0.001	1.2 ^{a} (0.0)	1.4 ^{b} (0.0)	1.6 ^{c} (0.0)	2.0 ^{d} (0.0)	<0.001
Riboflavin (mg)	1.7 ^{a} (0.0)	1.9 ^{a,b} (0.0)	1.8 ^{a,b} (0.0)	2.0 ^{b} (0.0)	<0.001	1.7 ^{a} (0.0)	1.8 ^{a,b} (0.0)	1.9 ^{b} (0.0)	2.1 ^{c} (0.0)	<0.001
Niacin (mg)	32.8 (0.5)	33.7 (0.5)	33.2 (0.5)	34.5 (0.5)	0.111	40.6 (0.3)	41.4 (0.3)	41.5 (0.3)	41.7 (0.4)	0.112
Folate (µg)	497 ^{a} (13)	620 ^{b} (12)	642 ^{b} (12)	746 ^{c} (13)	<0.001	469 ^{a} (7)	564 ^{b} (7)	624 ^{c} (7)	775 ^{d} (7)	<0.001
Magnesium (mg)	211 ^{a} (2)	235 ^{b} (2)	264 ^{c} (2)	309 ^{d} (2)	<0.001	273 ^{a} (2)	309 ^{b} (2)	345 ^{c} (2)	426 ^{d} (2)	<0.001
Potassium (mg)	2052 ^{a} (28)	2198 ^{b} (25)	2516 ^{c} (26)	2811 ^{d} (28)	<0.001	2304 ^{a} (18)	2637 ^{b} (17)	3006 ^{c} (17)	3680 ^{d} (18)	<0.001

Abbreviations: Q, quartile; SE, standard error. Different superscripts a, b, c, d denotes significant difference between groups ($p < 0.001$).
